# Age and regional disparity in HIV education among migrants in China: migrants population dynamic monitoring survey, 2014–2015

**DOI:** 10.1186/s12939-019-0999-x

**Published:** 2019-07-03

**Authors:** Zheng Zhu, Mengdi Guo, Darina V. Petrovsky, Tingyue Dong, Yan Hu, Bei Wu

**Affiliations:** 1 School of Nursing Fudan University, Shanghai, China; 2 Fudan University Centre for Evidence-based Nursing: A Joanna Briggs Institute Centre of Excellence, Shanghai, 200032 China; 3 School of public affairs, Zhejiang University, Hangzhou, China; 4 Rory Meyers College of Nursing, New York University, New York, 10010 USA; 5 NYU Aging Incubator, New York, 10010 USA; 6 Institute of Gerontology, Renmin University of China, Beijing, China

**Keywords:** HIV, AIDS, Migrant, HIV education, China, Migration

## Abstract

**Objective:**

A lack of education among migrants remains an important but overlooked issue that indirectly contributes to HIV transmission. It is necessary to know who has received HIV education and who has a lower probability of being educated among migrants across different regions and age groups in China.

**Methods:**

We used pooled data from the 2014 and 2015 Migrants Population Dynamic Monitoring Survey. The study population included 406,937 Chinese migrants. Participants were asked whether they had received any HIV education after migrating to the destination city. Regions were categorized into east-coast, central, northwest, southwest, west-Tibet, west-Uyghur, and northeast regions. Hierarchical logistic regression modeling was conducted to investigate the relationships between the independent variables and HIV education.

**Results:**

Of 406,937 participants, half (50.6%) had reported receiving HIV education. Individuals in the west-Uyghur region had the highest proportion of receiving HIV education (73.0%), followed by the southwest region (67.9%) and the west-Tibet region (54.8%). Methods of receiving HIV education varied among different age groups. Individuals who were in a region with a higher prevalence of HIV, a lower density of medical professionals, and a higher density of migrants were more likely to receive HIV education.

**Conclusions:**

The study showed significant regional disparities among migrants in China. More HIV resources need to be allocated to regions with large-scale floating populations, such as the east-coast region. Providing multiple options, including both new and traditional media, for both young and elderly migrants is essential. HIV education should be tailored to the age of migrants with low educational and income levels.

## Introduction

According to reports from the United Nations, there were 258 million international migrants and 740 million internal world migrants in 2017. The total number of migrants has increased by almost 50% since 2000, which means that in total, there are almost 1 billion people working or studying in a different place away from their hometown at the same time [1]. Pieke and Mallee (2014) described the internal migration in China as being one of the largest scale migrations in the world [2]. By 2016, the migrant population in China reached 282 million, which accounted for approximately 20% of the whole population in China [3].

Migrants have been identified as a high-risk group vulnerable to Human Immunodeficiency Virus (HIV) transmission [4]. For instance, a study of migrants in China showed that HIV prevalence increased among migrants from 0.032% in 2000 to 0.087% in 2011, compared to the 0.037% prevalence of HIV in the whole Chinese population during the same period [5]. In China, migrants are the target population in the HIV prevention strategy. Many social, economic and political factors contribute to the high risk of HIV infection among migrants, including restrictive health-related policies [6], lack of access to HIV education and services [6], discrimination and exploitation [7]. Among these factors, limited HIV knowledge among migrants remains an important but overlooked global issue that increases the risk of HIV transmission [8]. In China, low educational attainment among migrants makes them even more vulnerable to HIV transmission [9].

Previous studies have reported different proportions of migrants receiving HIV-prevention education in various populations in China [10, 11]. For instance, Yang et al. focused on male labor migrants in Xi’an city (northwest China) and found that over 40% of the participants failed to understand the concept of HIV prevention [10]. Wang et al. reported that approximately 70% of migrants in Hefei city (central China) had received HIV education and obtained knowledge of HIV prevention [11]. However, due to the scale of these studies, regional inequity has received much less direct attention. Additionally, China’s uneven regional development and cultural differences were the original sources of urban and rural divergence in HIV infection and mortality [12]. Since 1978, national public health system has experienced significant reconfigurations due to economic reforms. Currently, national policy still limits access to healthcare services in most rural areas. It remains unknown whether China’s regional variations are associated with the inequity of receiving HIV education.

With very few exceptions, existing research on HIV-prevention programs have mainly focused on young adults. However, in China the number and proportion of older migrants with HIV/AIDS have increased in recent years [13]. It is still unclear whether migrants’ age is associated with the proportion of migrants receiving HIV education. From the perspective of policy makers, more research is needed to elucidate which subpopulations would benefit the most from HIV education materials and identify age-appropriate methods of delivering HIV education.

To address this knowledge gap, we used data from a national population-based survey, the Migrant Population Dynamic Monitoring Survey (MDMS). The objectives of the study were to examine the rate and methods of HIV education by region and age group among migrants in China.

## Methods

### Sample

We used pooled data from the 2014 and 2015 MDMS Survey, a nationally representative demographic and health survey of migrant populations conducted annually by the Migrant Population Service Center of the People’s Republic of China (PRC). MDMS conducted a probability proportional to size (PPS) sampling method which is a stratified, multi-stage and proportional scale sampling. Additional details about the MDMS are available elsewhere [11]. Participation in the survey was restricted to migrants lived in the destination city for over 1 month. The final sample included 406,937 participants.

### Measures

#### Dependent variable

The primary outcome variable was whether a migrant has received HIV education after migrating to the current city (Yes or No). If the response was “Yes”, participants were asked to answer the following multiple choice question: “How did you access HIV education?” The eight choices were the following: lecture, book/magazine, media, face-to-face consultation, online medical consultation, community advocacy, bulletin board, and SMS/WeChat.

#### Covariates

##### Individual-level variables

We used four sociodemographic variables as covariates: age (continuous), gender (male or female), ethnicity (Han or minority), and marital status (single or otherwise). We also used four socioeconomic variables as covariates: education level (<middle school, high school or equivalent, college, and > college degree), monthly income (< 1500 CNY, 1500–5000 CNY, or > 5000 CNY), having a health record (“Yes” or “No”), an electronic medical record for both providers and patients that contains personal health information, including diagnosis, medications, and immunization record), and having basic medical insurance (“Yes” or “No”). In addition, we used four migrant-related variables as covariates. We collected information on reasons for migrating (working or studying, moving with family), length of migration (continuous) and type of household (rural or urban), and whether migrants had a 5-year long-term living preference (yes or otherwise).

##### Regional-level variables

We also used four regional-level variables: destination region (east-coast, central, northwest, southwest, west-Tibet, west-Uyghur, or northeast region), prevalence of HIV in each region (total number per 100,000 population), density of medical professionals (total number per 1000 population), and density of migrants (total number per 100,000 population). Destination region refers to the place to which participants migrated and was categorized based on regional economic growth reported by the 12th Five-year Plan of the Chinese government [12]. The data on HIV prevalence in each province/region were collected from China’s Notifiable Infectious Disease Reporting in 2015 [13]. The data on the density of medical professionals (total number per 1000 population) and the density of migrants (total number per 1000 population) in each region were obtained from 2015 Statistical Yearbook of China released by the National Bureau of Statistics of the PRC [14]. The regional division was based on regional economic growth reported by Chinese government [15].

### Statistical analysis

We used SPSS version 22.0 (IBM, Armonk, NY) to conduct the statistical analyses. Sampling weights were used for the survey so that the results would be representative of the migrant population. Descriptive statistical analyses were used to report demographic variables and methods of HIV education, including frequencies, percentages, means, and standard deviations. Chi-square analysis were conducted to determine statistical significance of differences in sociodemographic and socioeconomic characteristics and the rate of receiving HIV education among different regions. The study used a logistic regression model to investigate the predictions for receiving HIV education for migrants in different regions. The independent factors associated with receiving HIV education were entered into four models at each step. Model 1 included the main variables, including age and region. Model 2 included socioeconomic variables (gender, ethnicity, marital status, education level, monthly income, health-record status, and health-insurance status). Model 3 added prevalence of HIV, density of medical professionals, and density of migrants. Model 4, the final model, further included migrant-related variables such as reasons for migration, length of migration and type of household, and whether migrants reported having long-term living preferences. We considered a two-tailed *P* less than 0.05 as statistical significance in all analyses. Cox & Snell R^2^ measured the goodness of fit for logistic regression models.

We used HLM 7.0 to conduct hierarchical logistic regression modeling (HLM) to investigate the relationships between the independent variables and HIV education. HLM accommodates the variance among migrants in the same region, capturing the differences in statistics among regions as well as among migrants. Model 1 was the null model, which separated total variance into variance due to individual and regional levels. Model 2 was the random coefficient model, which included all the covariates at the individual level with a random effect. Model 3 included the intercepts and slopes as outcomes model. Log-likelihood (−2LL) measured the goodness of fit for logistic regression models.

## Results

### Sample characteristics

The study sample contained 406,937 migrants, including 173,985 (42.8%) migrants in the east-coast region, 67,986 (16.7%) in the central region, 41,994 (10.3%) in the northwest region, 55,995 (13.8%) in the southwest region, 17,982 (4.4%) in the west-Tibet region, 15,996 (3.9%) in the west-Uyghur region, and 32,999 (8.1%) in the northeast region (Table 1). In general, 44.2% of migrants were female, and 92.4% were Han Chinese. The average age was 35.2 (SD = 10.1), and the majority of the participants were between 19 and 35 (53.5%) years old. The average length of migration was 5.6 years (SD = 4.9). Most of the migrants came to the destination place for reasons of work or study (86.5%). Over half of the migrants (56.8%) had a preference for living in the destination place for over 5 years. Compared to the migrants in the central, western and northern regions, a greater number of migrants on the east-coast were female (45.2%) and younger (34.42 ± 9.57 years); they also had a higher monthly income (6864.46 ± 9547.64 CNY), and a lower proportion of having a health record (17.7%) and health insurance (88.3%). There was a statistically significant difference in all sociodemographic and socioeconomic variables (*P* = 0.000) across the regions.

**Table 1:** Demographic characteristics and outcomes by region among migrants

	All *n* = 406,937	East-coast *n* = 173,985 (42.8%)	Central *n* = 67,986 (16.7%)	Northwest *n* = 41,994 (10.3%)	Southwest *n* = 55,995 (13.8%)	West-Tibet n = 17,982 (4.4%)	West-Uyghur *n* = 15,996 (3.9%)	Northeast *n* = 32,999 (8.1%)
Gender								
Male	179,982 (44.2%)	78,707 (45.2%)	30,551 (44.9%)	18,263 (43.5%)	24,164 (43.2%)	7153 (39.8%)	6887 (43.1%)	14,257 (43.2%)
Female	226,955 (55.8%)	95,278 (54.8%)	37,435 (55.1%)	23,731 (56.5%)	31,831 (56.8%)	10,829 (60.2%)	9109 (56.9%)	18,742 (56.8%)
Age (years)	35.2 (10.1)	34.4 (9.6)	35.0 (9.7)	35.5 (10.0)	35.9 (10.66)	35.5 (10.7)	36.3 (10.6)	37.4(11.2)
15–18	6894 (1.7%)	2628 (1.5%)	1215 (1.8%)	650 (1.5%)	1097 (2.0%)	481 (2.7%)	310 (1.9%)	513 (1.6%)
19–35	217,713 (53.5%)	99,477 (57.2%)	36,081 (53.1%)	22,439 (53.4%)	28,148 (50.3%)	8948 (49.8%)	7558 (47.2%)	15,062 (45.6%)
36–60	177,806 (43.7%)	70,415 (40.5%)	30,284 (44.5%)	18,346 (43.7%)	25,849 (46.2%)	8352 (46.4%)	7927 (49.6%)	16,633 (50.4%)
60–95	4524 (1.1%)	1465 (0.8%)	406 (0.6%)	559 (1.3%)	901 (1.6%)	201 (1.1%)	201 (1.3%)	791 (2.4%)
Ethnicity								
Han	376,033 (92.4%)	166,158 (95.5%)	66,489 (97.8%)	38,355 (91.3%)	48,662 (89.6%)	12,119 (67.4%)	12,951 (81.0%)	31,299 (94.8%)
Minority	30,904 (7.6%)	7827 (4.5%)	1497 (2.2%)	3639 (8.7%)	7333 (13.1%)	5863 (32.6%)	3045 (19.0%)	1700 (5.2%)
Marital status								
Married	315,411 (77.5%)	136,794 (78.6%)	54,485 (80.1%)	34,492 (82.1%)	40,895 (73.0%)	12,357 (68.7%)	12,409 (77.6%)	23,979 (72.7%)
Otherwise	91,526 (22.5%)	37,191 (21.4%)	13,501 (19.9%)	7502 (17.9%)	15,100 (27.0%)	5625 (31.3%)	3587 (22.4%)	9020 (27.3%)
Educational attainment								
< Middle school	269,159 (66.1%)	113,612 (65.3%)	42,457 (62.4%)	28,585 (68.1%)	35,254 (63.0%)	14,110 (78.5%)	11,461 (71.6%)	23,680 (71.8%)
High school or equivalent	86,067 (21.1%)	35,792 (20.6%)	17,546 (25.8%)	8434 (20.1%)	12,698 (22.7%)	2992 (16.6%)	2626 (16.4%)	5979 (18.1%)
College	33,594 (8.3%)	15,073 (8.7%)	5759 (8.5%)	3446 (8.2%)	5222 (9.3%)	627 (3.5%)	1330 (8.3%)	2137 (6.5%)
> College degree	18,117 (4.4%)	9508 (5.5%)	2224 (3.3%)	1529 (3.6%)	2821 (5.1%)	253 (1.4%)	579 (3.7%)	1203 (3.7%)
Monthly income (CNY)	6052.7 (7792.7)	6864.5 (9547.6)	5639.1 (4778.7)	5273.4 (5537.1)	5549.4 (8135.9)	5961.7 (7029.4)	5370.9 (5878.6)	4851.3 (4412.8)
Having health record								
Yes	106,120 (26.1%)	30,708 (17.6%)	23,679 (34.8%)	12,666 (30.2%)	17,105 (30.5%)	6107 (34.0%)	4278 (26.7%)	11,577 (35.1%)
No	300,817 (73.9%)	143,277 (82.4%)	44,307 (65.2%)	29,328 (69.8%)	38,890 (69.5%)	11,875 (66.0%)	11,718 (73.3%)	21,422 (64.9%)
Having Basic Medical Insurance								
Yes	70,031 (17.2%)	44,858 (25.8%)	6584 (9.7%)	3104 (7.4%)	9664 (17.3%)	652 (3.6%)	1796 (11.2%)	3373 (10.2%)
No	336,906 (82.8%)	129,127 (74.2%)	61,402 (90.3%)	38,890 (92.6%)	46,331 (82.7%)	17,330 (96.4%)	14,200 (88.8%)	29,626 (89.8%)
Long-term residence inclination								
Yes	231,084 (56.8%)	95,127 (54.7%)	39,021 (57.4%)	24,290 (57.8%)	32,512 (58.1%)	7924 (44.1%)	10,284 (64.3%)	21,926 (66.4%)
Otherwise	175,853 (43.2%)	78,858 (45.3%)	28,965 (42.6%)	17,704 (42.2%)	23,483 (41.9%)	10,058 (55.9%)	5712 (35.7%)	11,073 (33.6%)
Length of Migration	5.6 (4.9)	5.5 (4.8)	5.2 (4.5)	6.0 (4.7)	5.5 (4.8)	5.4 (5.1)	6.5 (5.8)	6.7 (5.5)
Reason of Migration								
Working or studying	352,049 (86.5%)	157,048 (90.3%)	58,274 (85.7%)	33,131 (78.9%)	49,140 (87.8%)	14,848 (82.6%)	12,821 (80.2%)	26,787 (81.2%)
Moving with family	54,888 (13.5%)	16,937 (9.7%)	9712 (14.3%)	8863 (21.1%)	6855 (12.2%)	3134 (17.4%)	3175 (19.8%)	6212 (18.8%)
Type of household								
Rural	341,249 (83.9%)	146,783 (84.4%)	59,062 (86.9%)	36,434 (86.8%)	44,615 (79.7%)	15,963 (88.8%)	13,108 (81.9%)	25,284 (76.6%)
Urban	65,688 (16.1%)	27,202 (15.6%)	8924 (13.1%)	5560 (13.2%)	11,380 (20.3%)	2019 (11.2%)	2888 (18.1%)	7715 (23.4%)
Received HIV education	206,084 (50.6%)	79,916 (45.9%)	34,364 (50.5%)	18,735 (44.6%)	37,994 (67.9%)	9859 (54.8%)	11,675 (73.0%)	13,524 (41.0%)
Prevalence of HIV (total number per 100,000 population)*	37.7	28.3	31.8	17.2	130.3	32.0	168.8	23.3
Density of medical professionals (total number per 1000 population)*	5.9	6.2	5.4	6.3	5.5	5.4	6.9	5.8
Density of migrants (total number per 100,000)*	3315.5	4436.1	2368.9	3252.4	2687.1	2681.6	3092.5	2645.4

Individual data are n (%) or mean (SD). CNY=China Yuan Renminbi. * Prevalence of HIV, density of medical professionals, and density of migrants are regional data

### HIV education among migrants

**Fig. 1:**
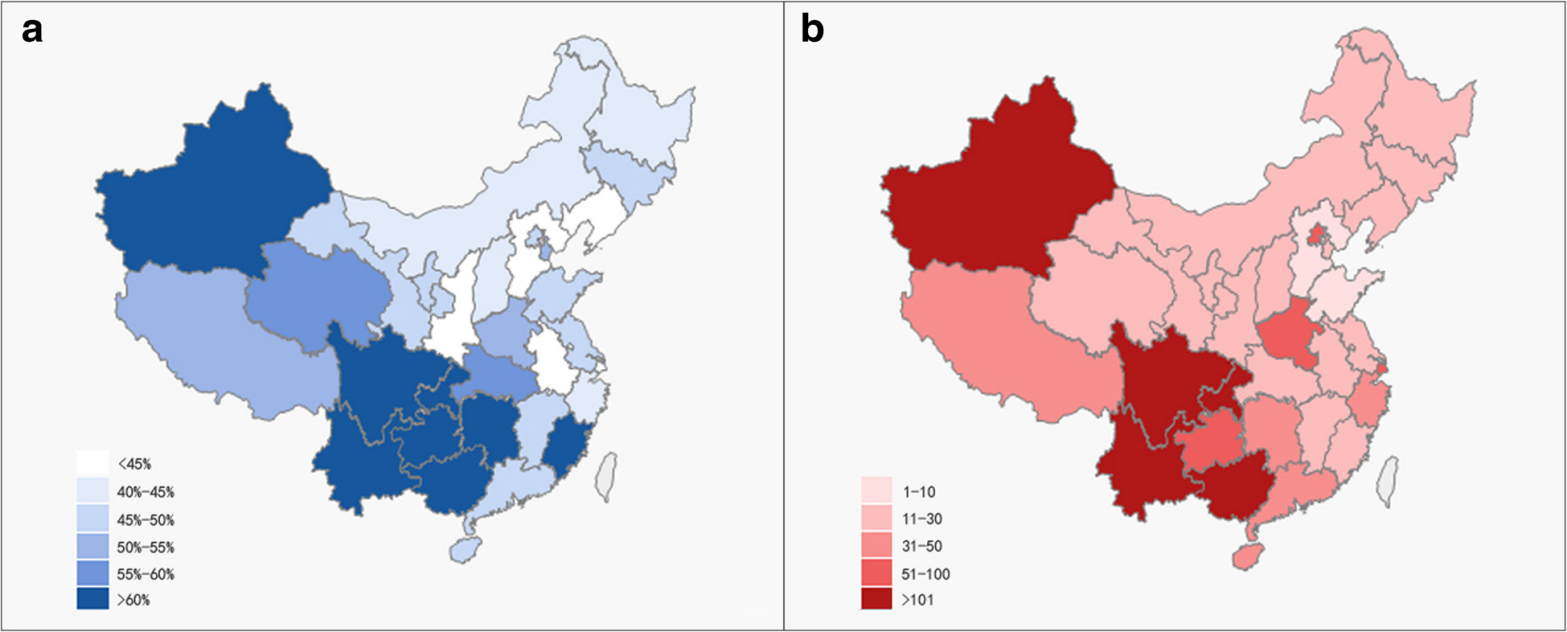
Proportion of receiving HIV education and prevalence of HIV in each province. **a** Proportion of receiving HIV education among migrants by province, pooled Migrants Population Dynamic Monitoring Survey Data 2014–2015 **b** Prevalence of HIV in each province (total number per 100,000 population), China’s Notifiable Infectious Disease Reporting in 2015

In the full sample, half of the participants (50.6%) reported receiving HIV education (Table 1). Compared to the migrants in the central and western regions, the rate of having received HIV education was significantly lower in the east-coast (45.9%) and northeast regions (41.0%). Figure 1(a) shows the map of the proportion of migrants in each region receiving HIV education. Among them, the west-Uyghur region had the highest proportion of migrants receiving HIV education (73.0%), followed by the southwest region (67.9%) and the west-Tibet region (54.8%). Figure 1(b) presents a map of HIV prevalence in each region. The west-Uyghur region has the highest prevalence of HIV (168.8 cases per 100,000 population), followed by the southwest region (130.3 cases per 100,000 population). In the southwest region, Yunnan Province (197.0 cases per 100,000 population) and Guangxi Province (153.0 cases per 100,000 population) had the highest prevalence of HIV.

**Fig. 2:**
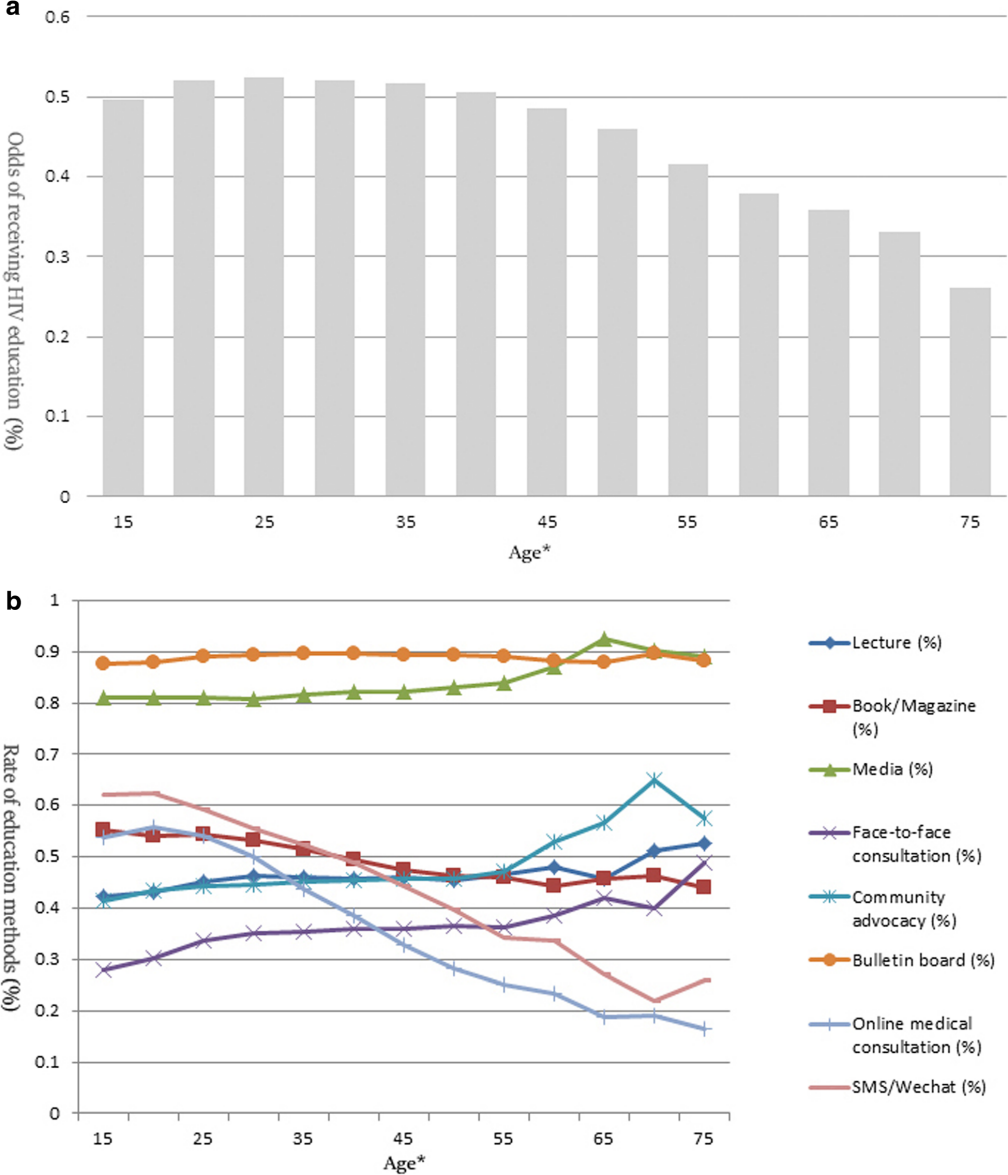
Proportion of receiving HIV education and education methods. **a** Proportion of receiving HIV education by age among migrants, pooled Migrants Population Dynamic Monitoring Survey Data 2014–2015; (**b**) Rate of education methods by age among migrants, pooled Migrants Population Dynamic Monitoring Survey Data 2014–2015; * Each group is plotted by at 5-year intervals, except the last interval is from 75 to 95 years old

In Fig. 2 (A), among 13 age groups, participants between 25 and 29 years old had the highest rate of receiving HIV education (52.5%), followed by participants were between 20 and 24 years old (52.0%), 30–34 years old (52.0%), and 35–39 years old (51.6%). In Fig. 2 (b), for the full sample, bulletin board (89.1%), media (86.1%), and SMS/WeChat (53.4%) were three most common methods for participants to receive HIV education. In the group under 35 years old, the three most common methods included a bulletin board (87.6–89.0%), media (80.8–81.1%), and SMS/WeChat (55.6–62.3%). In the 35 to 60-year-old group, the bulletin board was the most common method (89.1–89.6%), followed by media (82.0–84.0%) and books/magazine (46.2–49.5%). In the group over 60 years old, media was the most common method (87.1–92.5%), followed by bulletin board (88.1–89.8%) and community advocacy (52.9–64.9%).

### Multivariable and hierarchical logistic regression models

Table 2 shows the results of the multivariable logistic regression of factors associated with the proportion of migrants receiving HIV education in different regions (*P* = 0.000). In Model 3, after adjusting for the prevalence of HIV, density of medical professionals, and density of migrants, the OR values of the west-Uyghur region (OR = 2.479, CI: 2.329–2.629) and southwest region (OR = 1.977, CI = 1.889–2.066) decreased. In the final model, the west-Uyghur region had significantly higher proportion of receiving HIV education compared to other regions in China, followed by the southwest region.

**Table 2:** Factors related to receiving HIV education for migrants in different regions, pooled Migrants Population Dynamic Monitoring Survey Data 2014–2015

	Model 1		Model 2		Model 3		Model 4	
	OR (CI)	*P*	OR (CI)	*P*	OR (CI)	*P*	OR (CI)	*P*
Age	.990 (.989–.990)	.000	.990 (.990–.991)	.000	.990 (.990–.991)	.000	.990 (.990–.991)	.000
Region								
East-coast	1.187 (1.159–1.216)	.000	1.217 (1.187–1.247)	.000	1.228 (1.195–1.261)	.000	1.241 (1.208–1.275)	.000
Central	1.438 (1.400–1.477)	.000	1.349 (1.313–1.386)	.000	1.328 (1.292–1.365)	.000	1.339 (1.302–1.376)	.000
Northwest	1.138 (1.105–1.172)	.000	1.110 (1.077–1.143)	.000	1.159 (1.125–1.194)	.000	1.174 (1.139–1.209)	.000
Southwest	3.001 (2.917–3.087)	.000	2.959 (2.874–3.044)	.000	1.977 (1.889–2.066)	.000	2.004 (1.915–2.094)	.000
West-Tibet	1.717 (1.655–1.781)	.000	1.672 (1.610–1.735)	.000	1.526 (1.467–1.585)	.000	1.556 (1.496–1.617)	.000
West-Uyghur	3.862 (3.705–4.024)	.000	4.112 (3.939–4.285)	.000	2.479 (2.329–2.629)	.000	2.531 (2.378–2.684)	.000
Northeast	–	–	–	–	–	–	–	–

*Model 1 included age and region;

Model 2 added gender, ethnicity, marital status, education level, monthly income, health record status, and health insurance status;

Model 3 added prevalence of HIV (total number per 100,000 population), density of medical professionals (total number per 1000 population), and density of migration (total number per 100,000 population);

Model 4 added reasons of migration, length of migration, type of household, and whether or not have long-term living preference

**Model 1: P = 0.000 Cox & Snell R^2^ = 0.035; Model 2: P = 0.000 Cox & Snell R^2^ = 0.061; Model 3: P = 0.000 Cox & Snell R^2^ = 0.062; Model 4: *P* = 0.000 Cox & Snell R^2^ = 0.063;

Table 3 shows the results of the null model, random coefficient model, and slopes as outcomes model. Model 1 (null model) showed that the intraclass correlation coefficient was 3.29 (DEFF> 2); therefore, making it necessary to build the multilevel logistic regression model. Model 2 (random coefficient model) showed that after including 12 variables (age, gender, education, marital status, educational attainment, monthly income, having a health record, having insurance, reason for migration, length of migration, type of household, and long-term living preference), the average probability of receiving HIV education was still statistically higher than not receiving HIV education (OR = 2.953, *CI* = 2.748–3.173, *P* = 0.000). Parameter estimates on all included covariates were significant (*P* < 0.05). Individuals who were younger, male, minority, and married and who reported a higher monthly income, a college or above education, a health record, basic medical insurance, a shorter length of migration, and a long-term living preference were more likely to receive HIV education. In Model 3 (slopes as outcomes model), after adding individual-level and regional-level variables, the average probability of receiving HIV education remained statistically higher (OR = 4.635, *CI =* 4.045–5.310, *P* = 0.000). Individuals who were in a region with a higher prevalence of HIV, a lower density of medical professionals, and a higher density of migrants were more likely to receive HIV education (*P* < 0.000). After controlling for the remaining regional-level variables, the type of household did not significantly predict the rate of receiving HIV education among different regions (OR = 0.983, *CI* = .964–1.002, *P* = 0.076).

**Table 3:** Fixed effects estimates and random effect estimates for models of receiving HIV education, pooled Migrants Population Dynamic Monitoring Survey Data 2014–2015

	Model 1			Model 2			Model 3		
	OR	CI	*P*	OR	CI	*P*	OR	CI	*P*
Fixed Effects									
Intercept,γ_00_	1.026	1.020–1.032	.000	2.953	2.748–3.173	.000	4.635	4.045–5.310	.000
Individual level									
Age,β_1j_				.992	.992–.993	.000	.989	.989–.990	.000
Male (compared to female),β_2j_				1.043	1.029–1.057	.000	1.051	1.037–1.065	.000
Minority (compared to Han),β_3j_				1.344	1.314–1.378	.000	1.150	1.122–1.178	.000
Married (compared to otherwise),β_4j_				1.039	1.020–1.057	.000	1.088	1.068–1.107	.000
College or above (compared to otherwise),β_5j_				1.116	1.103–1.131	.000	1.108	1.094–1.122	.000
Monthly income,β_6j_				1.093	1.079–1.107	.000	1.139	1.124–1.153	.000
Having health record (compared to having no health record),β_7j_				1.962	1.934–1.988	.000	1.937	1.908–1.965	.000
Having basic medical insurance (compared to having no basic medical insurance),β_8j_				1.136	1.115–1.156	.000	1.176	1.154–1.198	.000
Urban Household, β_9j_				1.024	1.005–1.044	.016	.983	.964–1.002	.076
Reason of migration: working or studying (compared to otherwise),β_10j_				1.105	1.089–1.121	.000	1.112	1.095–1.129	.000
Length of migration,β_11j_				.996	.995–.998	.000	.914	.901–.928	.000
Having long-term preference over 5 years (compared to having no long-term preference over 5 year),β_12j_				1.137	1.115–1.156	.000	1.068	1.058–1.078	.000
Regional level									
Prevalence of HIV (total number per 100,000 population),γ_01_							1.009	1.008–1.009	.000
Density of medical professionals (total number per 1000 population),γ_02_							.906	.885–.928	.000
Density of migrants (total number per 100,000),γ_03_							1.000	.999–1.001	.629
Random Effects									
Intercept variance, ${\sigma}_u^2$	0.001			0.043			0.045		
Level 1 variance, ${\sigma}_e^2$	3.290			3.290			3.290		
-2LL	577,423.392			577,267.619			577,002.635		

## Discussion

To our knowledge, this is the first study using national population-based data to examine the proportion of migrant population receiving HIV education in China. The results indicated that the rates of receiving HIV education among seven regions in China were significantly different. Regions with a higher prevalence of HIV had a higher proportion of migrants receiving HIV-related education. The east-coast region with abundant healthcare and financial resources had a relatively lower rate of receiving HIV education compared to the central and most of the western regions. We also found that regional disparity in receiving HIV was associated with HIV prevalence rates in different regions. The method of receiving HIV education varied among different age groups.

According to *the Second Five-Year Action Plan to Control HIV/AIDS (2006–2010)* of the Chinese central government, one of the targets for the HIV-prevention education program was to achieve over 90% coverage among the migrant population before 2010 [16, 17]. However, in our study, the average proportion of patients receiving HIV education in different regions ranged from 41.0 to 73.0%, which largely fell behind the target of the five-year action plan. Due to the existing household registration system in China, it is difficult for migrants to obtain a permanent urban household registration in the destination city, especially in the more economically developed regions, such as the east-coast region [18, 19]. Without permanent household registration, migrants in China become marginalized and have no access to HIV education, public health services, and medical insurance. This phenomenon is more common in the east-coast region of China, which partially contributes to the regional disparity in the proportion of migrants receiving HIV education. A report from *the Lancet* has shown that regardless of how long migrants have lived in their destination cities in China, health services were still underused and inequitably distributed among migrants [20]. More importantly, there still exists a gap between the ongoing health-care reform and the growing challenge of migration [19, 21]. Changing governmental regulations regarding entitlements to medical services and prevention programs for migrants have increased the uncertainties of local authorities regarding policy implementation and put the migrant population at risk for HIV transmission.

We found that the regions with a higher density of medical professionals (total number per 1000 population) had a lower proportion of migrants receiving HIV education. In China, a higher density of medical professionals usually correlates with more abundant healthcare and financial resources in the region. This regional disparity can be explained in two ways. First, since China’s “Four Frees and One Care” policy was issued in 2003, the Chinese government started to offer free services, such as voluntary counseling and HIV testing, antiretroviral therapy, prevention of mother-to-child transmission, schooling for HIV/AIDS orphans, and care for people living with HIV [22]. These policies resulted in a significant increase in the investment in HIV-specialized services by the central government and its capacity for HIV prevention. Large-scale HIV prevention programs were also implemented in the provinces with the highest HIV prevalence, including the southwest region (e.g., Guangxi Province and Yunnan Province) and the west-Uygur Region [23]. From 2010 to 2015, the overall budget for HIV prevention in China was 280,923 billion CNY (44,344 billion USD) (Internal report, 2017). The central government allocated HIV-specialized prevention resources based on the level of HIV prevalence in each province, GDP, and annual income per capita, which meant that provinces with higher HIV prevalence and those that were less developed had more funding from the central government to conduct large-scale HIV prevention campaigns. However, current national HIV-specialized budget index from the central government does not include HIV prevalence among marginalized subgroups (i.e., LGBT, drug users, migrant populations) as key parameters for resource allocation, which directly contributes to the inequitable allocation of HIV resources among migrants in different regions. When HIV prevalence among these subgroups does not match the overall HIV distribution, HIV resources are not allocated to those who need them the most. As a result, migrants in regions with abundant financial and healthcare resources are less likely to receive HIV education due to these regions receiving less HIV-related funding from the central government.

Additionally, due to lower HIV prevalence in more developed regions, there is little effort to control and prevent HIV among migrant populations in these regions of China. As a result, although the total number of newly diagnosed HIV adults in these regions is relatively small, migrants still account for a large proportion of the newly diagnosed HIV cases in the more developed regions. For instance, in China, the Notifiable Infectious Disease Report showed that metropolitan areas densely populated by migrants, such as Beijing, Shanghai and Guangdong Province, accounted for 31 to 42% of the newly diagnosed patients in 2016 [16].

Inequitable HIV education allocation among migrants continues to exist globally. For instance, agencies of the United Nations, including the World Health Organization (WHO) and the Joint United Nations Programme on HIV/AIDS (UNAIDS), will continue their work to eliminate HIV transmission and increase service coverage in eastern and southern Africa over the next 5 years [24]. However, against the backdrop of the European migrant crisis, a 2014 report from UNAIDS noted that since 2004, in western and central Europe, migrants originating from high-prevalence countries accounted for over 15% of new HIV diagnoses, suggesting possible gaps in HIV prevention services for migrants [25]. More HIV education need to be allocated to regions with large-scale floating populations (migrants, immigrants, and refugees), such as central and northern Europe and central and south Asia.

Several other issues need to be considered when interpreting our findings. First, we found that older, female migrants with lower education level and lower monthly income were less likely to receive HIV education. Similarly, a study conducted in China showed that a lower level of education was associated with poorer HIV knowledge among male labor migrants [10]. The inequality of age and gender interacts with the possibility of accessing HIV education [26, 27]. Previous studies have shown that older adults do not have as much HIV knowledge as young people [28] and that there is also a rising prevalence of HIV among older adults [13]. In addition, women are more easily isolated from HIV intervention and healthcare services due to the mode of transmission and complex cultural factors [29]. There is a need to target education efforts for older and female migrants in China.

Second, we found that methods of receiving HIV education varied among age groups. Younger migrants reported receiving HIV education via online social media and online consultation platforms. In contrast, older migrants reported receiving HIV education through traditional media and face-to-face consultations. These results are consistent with previous studies in other countries indicating differences in how younger and older age groups received HIV education [30]. This can be explained in several ways. First, many young migrants may prefer online media and consultations to in-person physician visits, since online media can provide anonymity and are much more convenient for migrants to have access to latest information [31]. Second, older adults may face a number of barriers when adopting a new technology. Some of the challenges include not being able to physically use the device. In addition, older adults may not see the added value of using technology as opposed to mainstream media [32]. Since technologies are continually evolving, the government is constantly keeping pace with technological advancements to ensure the quality of health education. Younger migrants today may prefer to receive HIV education using newer technologies. However, there is still a large proportion of older migrants receiving HIV education through traditional media, including television, radio, and bulletin boards. Therefore, providing multiple streams of HIV education, including both new and traditional media, for both young and older migrants is essential.

### Limitations

Our study has several limitations. First, participants were asked only one question about whether they had received any HIV education after migrating to the destination city. The data were collected based on self-report, which could possibly contribute to reporting bias. Second, this was a cross-sectional study; therefore, we cannot tease out cause-and-effect relationships. The study, however, presents valuable findings for future longitudinal analyses of migrant populations.

## Conclusions

Our study generated new knowledge about factors associated with receiving HIV education among migrants in China. Our findings suggest significant regional disparities among migrants in China. Methods of receiving HIV education varied among different age groups. Our study findings suggest that health care resources need to be allocated to regions with large-scale migrant populations. The current funding allocation mechanisms in China may lead to an inequitable allocation of HIV resources among migrants in different regions. HIV education should be tailored to the age of migrants and should focus on female migrants with low educational and income levels.

## Data Availability

The datasets used during the current study are available from the corresponding author upon reasonable request.

## Abbreviations

AIDS: Acquired immune deficiency syndrome
HIV: The human immunodeficiency virus
MDMS: The Migrants Population Dynamic Monitoring Survey
PRC: The People’s Republic of China
SMS: Short message service
UNAIDS: The Joint United Nations Programme on HIV/AIDS
